# Pregnancy outcomes of monochorionic diamniotic and dichorionic diamniotic twin pregnancies conceived by assisted reproductive technology and conceived naturally: a study based on chorionic comparison

**DOI:** 10.1186/s12884-024-06521-z

**Published:** 2024-05-02

**Authors:** Shuhua Liu, Qianhua Xu, Jingyu Qian, Dehong Liu, Bin Zhang, Xianxia Chen, Mingming Zheng

**Affiliations:** 1 Department of Obstetrics and Gynecology, Hefei Maternal and Child Health Hospital, Hefei, 230001 China; 2 Department of Obstetrics and Gynecology, Anhui Women and Children’s Medical Center,, Hefei, 230001 China; 3 Department of Obstetrics and Gynecology, Maternal and Child Medical Center of Anhui Medical University, Hefei, 230001 China; 4 Reproductive Medicine Center, Department of Obstetrics and Gynecology, the First Affiliated Hospital of Anhui Medical University, Hefei, 230000 China

**Keywords:** Monochorionic twins, Dichorionic twins, Trichorionic triplets, Dichorionic triamniotic, ART

## Abstract

**Objective:**

To evaluate monochorionic diamniotic (MCDA) and dichorionic diamniotic (DCDA) twin pregnancies conceived by assisted reproductive technology (ART) and conceived naturally.

**Methods:**

We retrospectively analyzed the data on twin pregnancies conceived by ART from January 2015 to January 2022,and compared pregnancy outcomes of MCDA and DCDA twins conceived by ART with those of MCDA and DCDA twins conceived naturally, pregnancy outcomes between MCDA and DCDA twins conceived by ART, and pregnancy outcomes of DCT and TCT pregnancies reduced to DCDA pregnancies with those of DCDA pregnancies conceived naturally.

**Result:**

MCDA pregnancies conceived by ART accounted for 4.21% of the total pregnancies conceived by ART and 43.81% of the total MCDA pregnancies. DCDA pregnancies conceived by ART accounted for 95.79% of the total pregnancies conceived by ART and 93.26% of the total DCDA pregnancies. Women with MCDA pregnancies conceived by ART had a higher premature delivery rate, lower neonatal weights, a higher placenta previa rate, and a lower twin survival rate than those with MCDA pregnancies conceived naturally (all *p* < 0.05). Women with DCDA pregnancies conceived naturally had lower rates of preterm birth, higher neonatal weights, and higher twin survival rates than women with DCDA pregnancies conceived by ART and those with DCT and TCT pregnancies reduced to DCDA pregnancies (all *p* < 0.05).

**Conclusion:**

Our study confirms that the pregnancy outcomes of MCDA pregnancies conceived by ART are worse than those of MCDA pregnancies conceived naturally. Similarly, the pregnancy outcomes of naturally-conceived DCDA pregnancies are better than those of DCDA pregnancies conceived by ART and DCT and TCT pregnancies reduced to DCDA pregnancies.

## Introduction

Assisted reproductive technology (ART) has evolved into a well-established and advanced approach for addressing infertility.Over the past few decades, the incidence of multiple pregnancies has also gradually increased due to the use of ART [1–3]. Controlling the number of embryo transfer (ET) has reduced the incidence of multiple pregnancies to some extent [4, 5]. In China, the transfer of no more than two cleavage embryos and one blastocyst is recommended [6], however, multiple pregnancies remain inevitable. Our previous study showed that MCDA pregnancies accounted for 2.55% of all clinical pregnancies [7], while the DCT pregnancy rate accounted for 1.24% of all clinical pregnancies [8].

Twin pregnancies can be classified into MCDA, DCDA, and monochorionic monoamniotic (MCMA) pregnancies based on chorionic properties, among which MCDA pregnancies are defined as those in which twins share a common placenta, causing special materno-fetal complications, such as twin-twin transfusion syndrome (TTTS), twin anemia polycythemia sequence (TAPS), and selective intrauterine growth restriction (sIUGR). Due to infertile couples’ desires for twins [9], DCT and TCT triplet pregnancies are often reduced to DCDA twins by multifetal pregnancy reduction (MFPR) [10], so the incidence of monozygotic twin pregnancies conceived by ART is significantly higher than that of monozygotic twin pregnancies conceived naturally [11–15]. Evidence studies have shown that compared with naturally conceived pregnancies, multiple pregnancies conceived by ART are associated with an increased risk of obstetric complications, such as miscarriage [6, 11], preterm birth [16–22], perinatal mortality [22–24], low birth weight [17–20], and birth defects [17–20, 25, 26]. However, there are also inconsistent views on pregnancy outcomes regarding pregnancies conceived naturally and those conceived by ART. Some studies have shown that twin pregnancies conceived by ART have worse pregnancy outcomes than those conceived naturally [17–24]. However, in other studies, no difference in pregnancy outcomes was found between twin pregnancies conceived by ART and those conceived naturally [27–34]. Even one study showed that twin pregnancies conceived by ART had better pregnancy outcomes than those conceived naturally [35]. In addition, due to the low incidence of MCDA pregnancies conceived naturally and few reductions of DCT and TCT pregnancies to DCDA pregnancies, there are few studies comparing its extensive use with MCDA and DCDA conceived by ART and naturally conceived twins, and there are also inconsistent views. Given the above uncertain views, the aim of this study was to compare pregnancy outcomes of MCDA and DCDA pregnancies conceived by ART with those of MCDA and DCDA pregnancies conceived naturally, and to compare pregnancy outcomes of DCT and TCT pregnancies reduced to DCDA pregnancies with those of DCDA pregnancies conceived naturally.

## Materials and methods

We assessed data on twin pregnancies conceived by ART from January 2015 to January 2022 at the Reproductive Medicine Center, Department of Obstetrics and Gynecology, the First Affiliated Hospital of Anhui Medical University, and data on women with twin pregnancies who gave birth in the obstetrics department of the hospital. We excluded pregnant women with irregular obstetric examinations and incomplete data. The timing of termination of pregnancy was based on fetal and maternal conditions and was carried out in accordance with Chinese obstetric twin pregnancy guidelines. The termination of pregnancy was also carried out in accordance with the condition of the mother and the fetus, as well as the wishes of the couple. A total of 1951 women with DCDA pregnancies conceived by ART, 251 women with DCDA pregnancies conceived naturally, 92 women with MCDA pregnancies conceived by ART, 118 women with MCDA pregnancies conceived naturally, 125 women with TCT pregnancies reduced to DCDA pregnancies, and 16 women with DCT pregnancies reduced to DCDA pregnancies were included in this study. We compared pregnancy outcomes of MCDA and DCDA pregnancies conceived by ART with those of MCDA and DCDA pregnancies conceived by naturally, the outcomes between MCDA and DCDA pregnancies conceived by ART, and the outcomes of DCT and TCT pregnancies reduced to DCDA pregnancies with those of DCDA conceived naturally.

The cleavage embryos in the ART group were added 17 days to the day of ET, and blastocysts (added 19 days) to calculate the gestational age of birth, and to give an estimate of the last menstrual period (LMP).The numbers of cleavage embryos in the ART group at Day 17 and blastocysts (the number of blastocysts at Day 19) were added to calculate the gestational age at birth, giving an estimate of the LMP. The gestational age of twins conceived naturally was estimated by the date of the LMP, and if necessary, the gestational age of the embryo at the first trimester ultrasound. MCDA and DCDA pregnancies were diagnosed by transvaginal ultrasound, based on the “T-sign” or “lambda sign”, and chronicity was rechecked after delivery, using placental pathology if necessary. DCT and TCT reduction procedures were performed 6–8 weeks after ET and performed by experienced doctors at our Reproductive Medicine Center, using transvaginal ultrasound guidance to puncture and collect selected embryos without medication.

Due to the scarcity of twin pregnancies, we did not subdivide twin pregnancies conceived by ART into frozen embryos and fresh embryos to reanalyze the data. We considered the following variables: maternal age; gestational age; full-term delivery, premature delivery (subdivided into early preterm birth at 28–34 + 0 weeks and late preterm birth at 34–37 + 0 weeks); neonatal weight (subdivided into a low birth weight < 2500 g, very low birth weight < 1500 g, and fetal weight difference of > 25%); mode of delivery (subdivided into vaginal delivery and cesarean section); infant sex including male and female; Materno-fetal complications including gestational hypertension, gestational diabetes mellitus, intrahepatic cholestasis of pregnancy, premature rupture of membranes, placenta previa, postpartum hemorrhage (PPH), neonatal deformities, TTTS, and sIUGR; and the proportion of surviving fetuses (subdivided into singleton survival; twin survival; one live-born twin, one stillborn twin; and stillborn twins).

### Definitions used in the manuscript

Singleton survival means that one of the twins experienced spontaneous reduction before 20 weeks. One live-born and one stillborn twin means that one of the twins died in utero after 28 weeks of pregnancy. Stillborn twins means the both twins died in utero after 28 weeks of gestation. Full-term delivery refers to delivery after 37 weeks of pregnancy. Premature delivery indicates birth at less than 37 weeks gestation. The secondary sex ratio (SSR), defined as the proportion of live-born males out of all live births.

### Statistical analysis

Data conforming to the normal distribution are expressed as the mean ± standard deviation (SD). Categorical data were tested using chi-square or Fisher exact tests. The two-tailed t test was used for independent sample continuous variables. Data corresponding to the analysis were analyzed using the SPSS 24.0 software package (SPSS Inc., Chicago, IL). *P* < 0.05 was considered statistically significant. This study was approved by the Ethics Committee of the First Affiliated Hospital of Anhui Medical University.

## Results

### Pregnancy and maternal-infant outcomes of MCDA and DCDA pregnancies conceived naturally and by ART

A total of 1951 women with DCDA pregnancies conceived by ART and 251 with DCDA pregnancies conceived naturally were analyzed, and as shown in Table 1, the maternal age in the group with DCDA pregnancies conceived naturally was significantly lower than that in group with DCDA pregnancies conceived by ART (27.68 ± 3.15 years versus 29.53 ± 3.71 years, *p* < 0.001). The group with DCDA pregnancies conceived by ART was more likely to deliver prematurely than the group with DCDA pregnancies conceived naturally (48.2% versus 24.3%, *p* < 0.001), but there was no difference in the premature delivery rate between the subgroups (28–34 + 0 weeks, 34–37 + 0 weeks). In addition, the neonatal weight of the group with DCDA pregnancies conceived naturally was significantly higher than that of the group with DCDA pregnancies conceived by ART (3015.38 ± 681.42 g versus 2612.53 ± 523.61 g, *p* < 0.001). In terms of materno-fetal complications, the singleton survival rate of the group with DCDA pregnancies conceived by ART was significantly higher than that of group with DCDA pregnancies conceived naturally (14.92% versus 1.20%, *p* < 0.001), while the twin survival rate of the group with DCDA conceived naturally was higher (98.80% versus 84.47%, *p* < 0.001). There was no significant difference between the two groups regarding other maternal complications, except for rate of placenta previa (0.40% versus 2.97%, *p* < 0.001). In addition, there was no difference in the SSR of DCDA twins between ART and naturally conceived (*p* = 0.207).

**Table 1:** Pregnancy and maternal-infant outcomes of DCDA by ART and conceived naturally

	DCDA		
	By ART (*n* = 1951)	Conceived naturally (*n* = 251)	*P*
General Information			
Age of the pregnant woman (years)	29.53 ± 3.71	27.68 ± 3.15	**< 0.001**
gestational age (weeks)	36.20 ± 2.27	36.25 ± 1.83	0.701
pregnancy outcomes			
Full-term delivery (%)	1010/1951 (51.77)	190/251 (75.70)	**< 0.001**
Premature delivery (weeks) (%)	941/1951 (48.23)	61/251 (24.30)	
28–34 + 0	221/1951 (11.33)	15/251 (5.98)	0.844
34–37 + 0	720/1951 (36.90)	46/251 (18.33)	
Vaginal delivery (%)	175/1951 (8.97)	18/251 (7.17)	0.343
Cesarean section (%)	1776/1951 (91.03)	233/251 (92.83)	
Neonatal weight (g)	2612.53 ± 523.61	3015.38 ± 681.42	**< 0.001**
< 2500 g (%)	1293/3611 (35.81)	36/499 (7.33)	**< 0.001**
< 1500 g (%)	100/3611 (2.77)	4/499 (0.80)	**0.009**
Fetal weight difference>25%^a^ (%)	119/1660 (7.17)	20/248 (8.06)	0.613
Infant gender (%)			
Female	1614/3611 (44.70)	238/499 (47.70)	0.207
Male	1997/3611 (55.30)	261/499 (52.30)	
Maternal-infant outcomes (%)			
Gestational hypertension	105/1951 (5.38)	16/251 (6.37)	0.516
gestational diabetes mellitus	48/1951 (2.46)	7/251 (2.79)	0.754
intrahepatic cholestasis of pregnancy	8/1951 (0.41)	3/251 (1.20)	0.121
premature rupture of membranes	45/1951 (2.31)	6/251 (2.39)	0.934
placenta previa	58/1951 (2.97)	1/251 (0.40)	0.017
Postpartum hemorrhage	7/1951 (0.36)	2/251 (0.80)	0.274
Neonatal deformities	24/3611 (0.66)	2/499 (0.40)	0.762
discordant growth twin^b^	119/1660 (7.17)	20/251 (7.97)	0.649
Proportion of fetal survival types (%)			
Singleton survival^1^	291/1951 (14.92)	3/251 (1.20)	< 0.001
Twin survival	1648/1951 (84.47)	248/251 (98.80)	< 0.001
one live, one stillbirth^2^	0/1951	0/251	-
Twin stillbirths^3^	12/1951 (0.62)	0/251	0.382
SSR	1997/3611 (55.30)	261/499 (52.30)	0.207

Dichorionic twins: DCDA; Assisted reproductive technology: ART; The secondary sex ratio: SSR

^a^The proportion between twins

^b^Number of pregnancies

^1^Singleton survival means that one of the twins has spontaneous reduction before 20 weeks

^2^One Live, One Stillbirth means that one of the twins dies in utero after 28 weeks of pregnancy

^3^Twin stillbirths mean the death of twins in utero after 28 weeks of gestation

Bold values was statistically signifificant (*P* < 0.05)

Table 2 shows that for the pregnancy and maternal-infant outcomes of MCDA pregnancies, we included 118 women with MCDA pregnancies conceived naturally and 92 women with MCDA pregnancies conceived by ART. The two groups showed that the maternal age in the naturally conceived group was younger than that in the ART group (25.01 ± 2.08 years versus 29.47 ± 4.10 years, *p* < 0.001). The neonatal weight in the group with MCDA pregnancies conceived naturally was significantly higher than that in the group with MCDA pregnancies conceived by ART (2832.12 ± 624.51 g versus 2566.37 ± 507.27 g, *p* < 0.001), and the proportion of infants with a weight < 2500 g was significantly higher in the group with MCDA pregnancies conceived by ART (40.59% versus 17.18%, *p* < 0.001). The twin survival rate in the group with MCDA pregnancies conceived naturally was also significantly higher than that in the group with MCDA pregnancies conceived by ART (92.37% versus 81.52%, *p* = 0.018). The rate of singleton survival in the group with MCDA pregnancies conceived by ART was higher than that in the group with MCDA pregnancies conceived naturally (15.22% versus 6.78%, *p* = 0.048), suggesting that women with MCDA pregnancies conceived naturally were less likely to experience spontaneous reduction before 20 weeks. Similarly, the incidence of placenta previa was increased in the group with MCDA pregnancies conceived by ART (8.70% versus 0.85%, *p* = 0.011).

**Table 2:** Pregnancy and maternal-infant outcomes of MCDA by ART and conceived naturally

	MCDA		
	By ART (*n* = 92)	Conceived naturally (*n* = 118)	*P*
General Information			
Age of the pregnant woman (years)	29.47 ± 4.10	25.01 ± 2.08	*P* < 0.001
gestational age (weeks)	36.05 ± 2.30	35.99 ± 2.12	0.838
pregnancy outcomes			
Full-term delivery (%)	36/92 (39.13)	61/118 (51.69)	0.070
Premature delivery (weeks) (%)	56/92 (60.87)	57/118 (48.31)	
28–34 + 0	10/92 (10.87)	6/118 (5.08)	0.117
34–37 + 0	46/92 (50)	51/118 (43.22)	0.134
Vaginal delivery (%)	6/92 (6.52)	8/118 (6.78)	0.941
Cesarean section (%)	86/92 (93.48)	110/118 (93.22)	
Neonatal weight (g)	2566.37 ± 507.27	2832.12 ± 624.51	**< 0.001**
< 2500 g (%)	69/170 (40.59)	41/228 (17.98)	**< 0.001**
< 1500 g (%)	3/170 (1.76)	3/228 (1.32)	0.704
Fetal weight difference>25%^a^ (%)	5/78 (6.41)	7/110 (6.36)	NS
Infant gender (%)			
Female	70/170 (41.18)	112/228 (49.12)	0.115
Male	100/170 (58.82)	116/228 (50.88)	
Maternal-infant outcomes (%)			
Gestational hypertension	8/92 (8.70)	11/118 (9.32)	0.875
gestational diabetes mellitus	5/92 (5.43)	9/118 (7.63)	0.527
intrahepatic cholestasis of pregnancy	2/92 (2.17)	4/118 (3.39)	0.698
premature rupture of membranes	5/92 (5.43)	7/118 (5.93)	0.878
placenta previa	8/92 (8.70)	1/118 (0.85)	**0.011**
Postpartum hemorrhage	1/92 (1.09)	4/118 (3.39)	0.338
Neonatal deformities	1/92 (1.09)	0/118 (0)	0.438
twin-twin transfusion syndrome	4/92 (4.35)	8/118 (6.78)	0.415
selective intrauterine growth restriction^b^	5/92 (5.43)	7/118 (5.93)	0.878
Proportion of fetal survival types (%)			
Singleton survival^1^	14/92 (15.22)	8/118(6.78)	**0.048**
Twin survival	75/92 (81.52)	109/118 (92.37)	**0.018**
one live, one stillbirth^2^	3/92 (3.26)	1/118 (0.85)	0.321
Twin stillbirths^3^	0/92	0/118	-
SSR	100/170 (58.82)	116/228 (50.88)	0.115

Monochorionic twins: MCDA; Assisted reproductive technology: ART; The secondary sex ratio: SSR

^a^The proportion between twins

^b^Number of pregnancies

^1^Singleton survival means that one of the twins has spontaneous reduction before 20 weeks

^2^One Live, One Stillbirth means that one of the twins dies in utero after 28 weeks of pregnancy

^3^Twin stillbirths mean the death of twins in utero after 28 weeks of gestation

Bold values was statistically signifificant (*P* < 0.05)

### Pregnancy and maternal-infant outcomes of MCDA and DCDA pregnancies conceived by ART

As shown in Table 3, The rate of premature delivery in the group with MCDA pregnancies conceived by ART was significantly higher than that in the group with DCDA pregnancies conceived by ART (60.87% versus 48.23%, *p* = 0.018), especially between 34 and 37 + 0 weeks (50% versus 36.90%, *p* = 0.011). In terms of maternal-infant complications, we observed slightly worse pregnancy outcomes in the MCDA group, such as a higher incidence of placenta previa in the MCDA group than in the DCDA group (8.70% versus 2.97%, *p* = 0.008) and a higher rate of stillbirth of one of the twins (6.26% versus 0, *p* < 0.001).

**Table 3:** Pregnancy and maternal-infant outcomes of MCDA and DCDA by ART

	ART		
	DCDA (*n* = 1951)	MCDA (*n* = 92)	*P*
General Information			
Age of the pregnant woman (years)	29.53 ± 3.71	29.47 ± 4.10	0.879
gestational age (weeks)	36.20 ± 2.27	36.05 ± 2.30	0.542
pregnancy outcomes			
Full-term delivery (%)	1010/1951 (51.77)	36/92 (39.13)	**0.018**
Premature delivery (weeks) (%)	941/1951 (48.23)	56/92 (60.87)	
28–34 + 0	221/1951 (11.33)	10/92 (10.87)	0.892
34–37 + 0	720/1951 (36.90)	46/92 (50)	**0.011**
Vaginal delivery (%)	175/1951 (8.97)	6/92 (6.52)	0.419
Cesarean section (%)	1776/1951 (91.03)	86/92 (93.48)	
Neonatal weight (g)	2612.53 ± 523.61	2566.37 ± 507.27	0.263
< 2500 g (%)	1293/3611 (35.81)	69/170 (40.59)	0.204
< 1500 g (%)	100/3611 (2.77)	3/170 (1.76)	0.628
Fetal weight difference>25%^a^ (%)	119/1660 (7.17)	5/78 (6.41)	0.799
Infant gender			
Female (%)	1614/3611 (44.70)	70/170 (41.18)	0.367
Male (%)	1997/3611 (55.30)	100/170 (58.82)	
Maternal-infant outcomes (%)			
Gestational hypertension	105/1951 (5.38)	8/92 (8.70)	0.174
gestational diabetes mellitus	48/1951 (2.46)	5/92 (5.43)	0.087
intrahepatic cholestasis of pregnancy	8/1951 (0.41)	2/92 (2.17)	0.071
premature rupture of membranes	45/1951 (2.31)	5/92 (5.43)	0.071
placenta previa	58/1951 (2.97)	8/92 (8.70)	**0.008**
Postpartum hemorrhage	7/1951 (0.36)	1/92 (1.09)	0.309
Neonatal deformities	24/3611 (0.66)	2/170 (1.18)	0.328
twin-twin transfusion syndrome	-	4/92	-
selective intrauterine growth restriction	-	5/92	-
discordant growth twin^b^	119/1660	-	-
Proportion of fetal survival types (%)			
Singleton survival^1^	291/1951 (14.92)	14/92 (15.22)	0.937
Twin survival	1648/1951 (84.47)	75/92 (81.52)	0.447
one live, one stillbirth^2^	0/1951 (0)	3/92 (3.26)	*P* < 0.001
Twin stillbirths^3^	12/1951 (0.62)	0/92 (0)	NS
SSR	1997/3611 (55.30)	100/170 (58.82)	0.367

Dichorionic twins: DCDA; Monochorionic twins: MCDA; Assisted reproductive technology: ART; The secondary sex ratio: SSR

^a^The proportion between twins

^b^Number of pregnancies

^1^Singleton survival means that one of the twins has spontaneous reduction before 20 weeks

^2^One Live, One Stillbirth means that one of the twins dies in utero after 28 weeks of pregnancy

^3^Twin stillbirths mean the death of twins in utero after 28 weeks of gestation

Bold values was statistically signifificant (*P* < 0.05)

**Pregnancy and maternal-infant outcomes of DCDA pregnancies conceived naturally and DCT and TCT pregnancies reduced to DCDA**.

Based on our previous studies, TCT and DCT triplets reduction to singleton pregnancy.had the best pregnancy outcomes [6].However, there are also pregnant patients who wish to retain twin pregnancies, and for DCT pregnancies, we reduced one of the MCDA twins.

From Table 4, we can see that pregnant women in the group with DCDA pregnancies conceived naturally were younger than those in the group with TCT pregnancies reduced to DCDA pregnancies (27.68 ± 3.148 years versus 29.54 ± 3.458 years, *p* < 0.001). In terms of pregnancy outcomes, premature delivery rates were higher in the group with TCT pregnancies reduced to DCDA pregnancies than in the group with DCDA pregnancies conceived naturally and were highest in the premature delivery subgroup (28–34 + 0 weeks, 34–37 + 0 weeks) (*p* = 0.002, *p* = 0.003, respectively). Similarly, the neonatal weight of the group with DCDA pregnancies conceived naturally was significantly higher than that in the group with TCT pregnancies reduced to DCDA pregnancies (3015.38 ± 681.42 g versus 2567.66 ± 571.38 g, *p* < 0.001), and the proportion of lower neonatal weight (< 2500 g, < 1500 g, respectively) in the group with TCT pregnancies reduced to DCDA pregnancies was also higher (39.15% versus 7.21%, *p* < 0.001; 4.68% versus 0.80%, *p* = 0.001). Pregnancy outcomes after TCT pregnancy reduction were also improved, with no difference in the probability of maternal complications for women with DCDA pregnancies conceived naturally (all *P* > 0.05). However, the twin survival rate was higher in the group with DCDA pregnancies conceived naturally (98.80% versus 86.40%, *p* < 0.001), and the singleton survival rate was increased in the group with TCT pregnancies reduced to DCDA pregnancies (11.20% versus 1.20%, *p* < 0.001), which indicated that the probability of spontaneous reduction after TCT reduction was also higher.

**Table 4:** Pregnancy and maternal-infant outcomes of DCDA conceived naturally and TCT reduced to DCDA

	DCDA		
	TCT reduced to DCDA (*n* = 125)	Conceived naturally (*n* = 251)	*P*
General Information			
Age of the pregnant woman (years)	29.54 ± 3.458	27.68 ± 3.148	*p* < 0.001
gestational age (weeks)	35.84 ± 2.381	36.25 ± 1.83	0.091
pregnancy outcomes			
Full-term delivery (%)	65/125 (52)	190/251 (75.70)	*p* < 0.001
Premature delivery(weeks) (%)	60/125 (48)	61/251 (2.39)	
28–34 + 0	20/125 (16)	15/251 (5.98)	**0.002**
34–37 + 0	40/125 (32)	46/251 (18.33)	**0.003**
Vaginal delivery (%)	8/125 (6.4)	18/251 (7.17)	0.781
Cesarean section (%)	117/125 (93.6)	233/251 (92.83)	
Neonatal weight (g)	2567.66 ± 571.38	3015.38 ± 681.42	*p* < 0.001
< 2500 g (%)	92/235 (39.15)	36/499 (7.21)	*p* < 0.001
< 1500 g (%)	11/235 (4.68)	4/499 (0.80)	**0.001**
Fetal weight difference>25%^a^ (%)	9/110 (8.18)	20/248 (8.06)	0.970
Infant gender			
Female (%)	117/235 (49.79)	238/499 (47.70)	0.597
Male (%)	118/235 (50.21)	261/499 (52.30)	
Maternal-infant outcomes (%)			
Gestational hypertension	3/125 (2.4)	16/251 (6.37)	0.097
gestational diabetes mellitus	1/125 (0.8)	7/251 (2.79)	0.278
intrahepatic cholestasis of pregnancy	1/125 (0.8)	3/251 (1.20)	NS
premature rupture of membranes	4/125 (3.2)	6/251 (2.39)	0.736
placenta previa	0/125 (0)	1/251	NS
Postpartum hemorrhage	1/125 (0.8)	2/251 (0.80)	NS
Neonatal deformities	4/235 (0.43)	2/499 (0.40)	0.087
discordant growth twin^b^	9/125 (7.2)	20/251 (7.97)	0.793
Proportion of fetal survival types (%)			
Singleton survival^1^	14/125 (11.20)	3/251 (1.20)	**< 0.001**
Twin survival	108/125 (86.40)	248/251 (98.80)	**< 0.001**
one live, one stillbirth^2^	2/125 (1.6)	0/251 (0)	0.110
Twin stillbirths^3^	1/125 (0.8)	0/251 (0)	0.332
SSR	118/235 (50.21)	261/499 (52.30)	0.597

Dichorionic twins: DCDA; Trichorionic triplets: TCT; The secondary sex ratio: SSR

^a^The proportion between twins

^a^The proportion between twins

^b^Number of pregnancies

^1^Singleton survival means that one of the twins has spontaneous reduction before 20 weeks

^2^One Live, One Stillbirth means that one of the twins dies in utero after 28 weeks of pregnancy

^3^Twin stillbirths mean the death of twins in utero after 28 weeks of gestation

Bold values was statistically signifificant (*P* < 0.05)

The results of the comparison between the groups with DCT pregnancies reduced to DCDA pregnancies and naturally conceived pregnancies are shown in Table 5. The maternal age in the group with DCT pregnancies reduced to DCDA pregnancies was significantly higher (29.88 ± 4.288 years versus 27.68 ± 3.148 years, *p* = 0.009). The neonatal weight in the group with DCT pregnancies reduced to DCDA pregnancies was lower than that in the group with DCDA pregnancies conceived naturally (2734.09 ± 615.55 g versus 3015.38 ± 681.42 g, *p* < 0.001), and the proportion of neonates with a weight < 2500 g was higher than that in the group with DCDA pregnancies conceived naturally (40.91% versus 7.21%, *p* < 0.001).However, singleton survival accounted for a higher proportion than DCDA twin survival in the naturally conceived group (62.50% versus 3.19%, *p* < 0.001), which also suggests an increased incidence of spontaneous reduction following MFPR.

**Table 5:** Pregnancy and maternal-infant outcomes of DCDA conceived naturally and DCT reduced to DCDA

	DCDA		
	DCT reduced to DCDA(*n* = 16)	Conceived naturally (*n* = 251)	*P*
General Information			
Age of the pregnant woman (years)	29.88 ± 4.288	27.68 ± 3.148	**0.009**
gestational age (weeks)	37.187 ± 2.90	36.25 ± 1.827	0.221
pregnancy outcomes			
Full-term delivery (%)	9/16 (56.25)	190/251 (75.70)	0.133
Premature delivery (weeks (%)	7/16 (43.75)	61/251 (2.39)	
28–34 + 0	1/16 (6.25)	15/251 (5.98)	NS
34–37 + 0	6/16 (37.5)	46/251 (18.33)	0.095
Vaginal delivery (%)	3/16 (18.75)	18/251 (7.17)	0.120
Cesarean section (%)	13/16 (81.25)	233/251 (92.83)	
Neonatal weight (g)	2734.09 ± 615.55	3015.38 ± 681.42	**< 0.001**
< 2500 g (%)	9/22 (40.91)	36/499 (7.21)	**< 0.001**
< 1500 g (%)	1/22 (4.55)	4/499 (0.80)	0.195
Fetal weight difference>25%^a^ (%)	0/6 (0)	20/248 (8.06)	0.588
Infant gender			
Female (%)	12/22 (54.55)	238/499 (47.70)	0.529
Male (%)	10/22 (45.45)	261/499 (52.30)	
Maternal-infant outcomes (%)			
Gestational hypertension	0/16 (0)	16/251 (6.37)	0.608
gestational diabetes mellitus	0/16 (0)	7/251 (2.79)	NS
intrahepatic cholestasis of pregnancy	0/16 (0)	3/251 (1.20)	NS
premature rupture of membranes	0/16 (0)	6/251 (2.39)	NS
placenta previa	0/16 (0)	1/251	NS
Postpartum hemorrhage	0/16 (0)	2/251 (0.80)	NS
Neonatal deformities	0/16 (0)	2/499 (0.40)	NS
discordant growth twin^b^	0/16 (0)	20/251 (7.97)	0.618
Proportion of fetal survival types (%)			
Singleton survival^1^	10/16 (62.50)	3/251 (1.20)	*p* < 0.001
Twin survival	6/16 (37.5)	248/251 (98.80)	0.643
one live, one stillbirth^2^	0/16 (0)	0/251 (0)	NS
Twin stillbirths^3^	0/16 (0)	0/251 (0)	-
SSR	10/22 (45.45)	261/499 (52.30)	0.529

Dichorionic twins: DCDA; Dichorionic triamniotic: DCT; The secondary sex ratio: SSR

^a^The proportion between twins

^a^The proportion between twins

^b^Number of pregnancies

^1^Singleton survival means that one of the twins has spontaneous reduction before 20 weeks

^2^One Live, One Stillbirth means that one of the twins dies in utero after 28 weeks of pregnancy

^3^Twin stillbirths mean the death of twins in utero after 28 weeks of gestation

Bold values was statistically signifificant (*P* < 0.05)

## Discussion

As we all know, the risk of multiple pregnancies is significantly higher than that of singleton pregnancies. In twin pregnancies, DCDA twin pregnancies were associated with significantly better outcomes than MCDA twin pregnancies.Previous reports have shown that twin pregnancies account for approximately 1.5% of all naturally conceived pregnancies but only 15–30% of pregnancies conceived by ART [31, 33, 34]. According to the CSRM guidelines, several fetal reduction committees of the Chinese Society of Reproductive Studies [36], physicians should recommend that couples reduce the number of fetuses they have and preserve singleton pregnancies as much as possible.However, through this investigation, it was found that MCDA pregnancies conceived by ART accounted for 4.21% of the total twin pregnancies conceived by ART and 43.81% of the total MCDA pregnancies, and DCDA pregnancies conceived by ART accounted for 95.79% of the total twin pregnancies conceived by ART and 93.26% of the total DCDA pregnancies. There are many factors that can be involved in multiple pregnancies, among which the age of the mother plays a crucial role. Previous studies have noted that women who are older are more likely to have monozygotic twins [37, 38]. Similarly, we also found that the maternal age of women who conceived twins by ART was generally higher than that of women who conceived twins naturally.As we are a single-center study, the number of cases of MCDA twin is limited.In this study, we recruited fewer MCDA twins conceived by ART than naturally conceived MCDA twins because some MCDA twins were reduced to single fetuses, and some had spontaneous abortions in the first trimester.

We know that twins themselves have many complications compared to singletons, especially the MCDA twins.Through this study, we confirmed that the pregnancy outcomes of MCDA pregnancies are worse than those of DCDA pregnancies, regardless of the mode of conception. In addition, The study showed that maternal complications of twin pregnancies conceived by ART were comparable to those of twin pregnancies conceived naturally, except that the incidence of placenta previa was higher in the ART group than in the group with natural conception. The association mechanism of ART in increasing the incidence of placenta previa remains uncertain [39–41], even after controlling for maternal age [42], the number of embryos [40, 43], and subfertility factors [44, 45]. However, it has been suggested that it may be related to altered endometrial blood flow, placental abnormalities [46]. In addition, it has been suggested that the mechanism of low placental implantation during ET and the induction of uterine contractions after ET caused by the cervical catheter is considered to be the mechanism of low placental implantation [45]. Another study also reported that factors related to the ART procedure itself, including the composition of the medium, the duration of embryo culture, the procedure in which the embryos are frozen and thawed, the potential for polyspermic fertilization, delays in oocyte fertilization, changes in the hormonal environment at the time of ET, the manipulation of gametes and embryos, or a combination of these factors, may increase the risk of placenta previa [47, 48].

In addition, this survey shows that the rate of premature delivery of DCDA pregnancies conceived by ART and TCT reduction was higher than that of DCDA pregnancies conceived naturally, and the materno-fetal outcome is also worse. This result is as expected, and as our previous research reported, which shown that the rate of miscarriage and preterm birth increased after TCT pregnancy reduction [6].Again, as we expected, the rate of premature delivery was higher for both MCDA pregnancies conceived naturally and by ART than for DCDA pregnancies, which is basically consistent with previous research [49, 50]. However, the results suggest that there is no difference in the rate of premature delivery between DCT pregnancies reduced to DCDA pregnancies and naturally conceived pregnancies, but higher spontaneous reduction in the DCT reduction group.This may be related to a small number of cases, but it can at least suggest that DCT pregnancies reduced to DCDA pregnancies have worse maternal-infant outcomes than DCDA pregnancies conceived naturally. In additon, the neonatal weight of the group with DCDA pregnancies conceived naturally was higher than that of the group with DCDA pregnancies conceived by ART and TCT and DCT pregnancies with reduction, the neonatal weight of the group with MCDA pregnancies conceived naturally was higher than that of the group with MCDA pregnancies conceived by ART, and the incidence of neonates with a weight < 2500 g was higher in the group conceived by ART, which is consistent with the findings of Trojner Bregar A et al. [51]. There was no significant difference in neonatal weight between the groups with MCDA and DCDA pregnancies conceived by ART, but from the perspective of neonatal weight in these two groups, the neonatal weight of both groups was still low. The proportion of neonates with a weight < 1500 g was only higher in the group with TCT pregnancies reduced to DCDA pregnancies than in the group with DCDA pregnancies conceived naturally, and there was almost no difference in the other groups. This shows that MFPR has an impact in the pregnancy as have seen several papers.

Spontaneous reduction is also a common complication of ART and multiple pregnancy [52]. The rate of spontaneous reduction in DCDA pregnancies conceived by in vitro fertilization (IVF) has previously been reported to be between 5 and 50% [53–56]. Some studies have noted that spontaneous reduction easily increases the chance of miscarriage, premature birth, and low birth weight for the remaining fetus [6, 57, 58]. The same problem was found in our study.Some studies noted that spontaneous reduction after ART may be influenced by factors such as embryo quality, maternal age, injury, infection, inflammation, and hormonal changes [51, 57–61].

It is reassuring to note that in the current state, our ART procedures do not affect the sex ratio law in the naturally conceived state. This is different from previous reports, and studies have pointed out that ART has a certain impact on the sex ratio [62–64].Several studies on the sex ratio of the Chinese population have shown that the sex ratio of the population has gradually become normal in recent years [65, 66].but we would like to continue to study the clinical data of our newborns in future work to exclude one-sided conclusions due to the insufficient amount of data in this study.

In our study, the mother-infant outcomes of twins conceived by assisted reproduction were similar to those of spontaneously conceived twins, which were largely similar to the results of several previous studies, except for the incidence of placenta previa described above [28, 33, 35, 67]. However, studies have shown that the rate of maternal complications are higher in twin pregnancies conceived by ART than in those conceived naturally [68–70], and the rate of neonatal defects increases significantly in twin pregnancies conceived by ART [71, 72].The stillbirth rate differs significantly between MCDA and DCDA pregnancies conceived by ART, which is well known that the perinatal outcome of MCDA twins depends primarily on the presence or absence of specific complications, such as TTTS.

Finally, this study has certain limitations. Because our hospital is a tertiary hospital, patients before 12 weeks of pregnancy generally visit our community hospital for prenatal examination and registration because the examination methods and diagnostic ability of community hospitals may have certain deficiencies; for the diagnosis of ambiguous chorionic diseases, we may need pathological assistance to clarify diagnoses after delivery. We also did not assess spontaneous fetal miscarriage in the first trimester of pregnancy. However, we analyzed the type of fetal survival. In this regard, the results can also be reflected from this point of view. Based on previous research, DCT pregnancies in our center are mostly reduced to DCDA pregnancies, resulting in less data, and we hope to combine multicenter data for comprehensive analysis in the future.Due to the retrospective analysis, some patients have vague recollection of assisted reproductive technology (IVF or ICSI), so we did not make perfect statistics for this part of the data, and the analysis of SSR may not be comprehensive enough. However, our study is also somewhat advantageous, as we have an almost comprehensive analysis of pregnancy outcomes for several types of twin pregnancies, Such as, comparing pregnancy outcomes of DCT and TCT pregnancies reduced to DCDA pregnancies with those of DCDA pregnancies conceived naturally, which is in addition to the very little literature available.

## Conclusions

It is well established that DCDA twin pregnancies have better outcomes than MCDA twin pregnancies.In this study, We confirm that pregnancy outcomes of MCDA pregnancies conceived by ART are worse than those of MCDA pregnancies conceived naturally.Similarly, the pregnancy outcomes of naturally-conceived DCDA pregnancies are better than those of DCDA pregnancies conceived by ART and DCT and TCT pregnancies reduced to DCDA pregnancies. However, the causes and mechanisms of this aspect are not specifically described. Through this conclusion, we will continue to study the questions raised in the article.

## Data Availability

The datasets used and/or analyzed during the current study are also available from the corresponding author on reasonable request.

## Abbreviations

MCDA: monochorionic-diamniotic
DCDA: dichorionic-diamniotic
ART: assisted reproductive technology
DCT: dichorionic triamniotic triplet pregnancy
TCT: trichorionic triamniotic triplet pregnancy
TTTS: twin-twin transfusion syndrome
TAPS: twin anemia polycythemia sequence
sIUGR: selective intrauterine growth restriction
MFPR: multifetal pregnancy reduction
LMP: the last menstrual period; ET: embryo transfer
PPH: postpartum hemorrhage
IVF: in vitro fertilization
SD: mean ± standard deviation
